# Supplementary material for: A role for subducted albite in the water cycle and alkalinity of subduction fluids
Source: Nat Commun. 2021 Feb 19;12:1155. doi: 10.1038/s41467-021-21419-6 (PMC7895919; doi:10.1038/s41467-021-21419-6)
Supplement: Supplementary file 2 — Supplementary Information [file 41467_2021_21419_MOESM2_ESM.pdf]

Supplementary Information

A role for subducted albite in the water cycle and alkalinity of subduction fluids

Gil Chan Hwang[1,±], Huijeong Hwang[1,±], Yoonah Bang[1,±], Jinhyuk Choi[1], Yong Park[2], Tae-Yeol Jeon[3], Boknam Chae[3], Haemyeong Jung[2], Yongjae Lee[1,*]

[1] Department of Earth System Sciences, Yonsei University, Seoul 03722, Korea

[2] School of Earth and Environmental Sciences, Seoul National University, Seoul 08826, Korea

[3] Beamline Science Division, Pohang Accelerator Laboratory, Pohang 37673, Korea

[*]Corresponding author Email: yongjaelee@yonsei.ac.kr

[±]These authors contributed equally to this work.

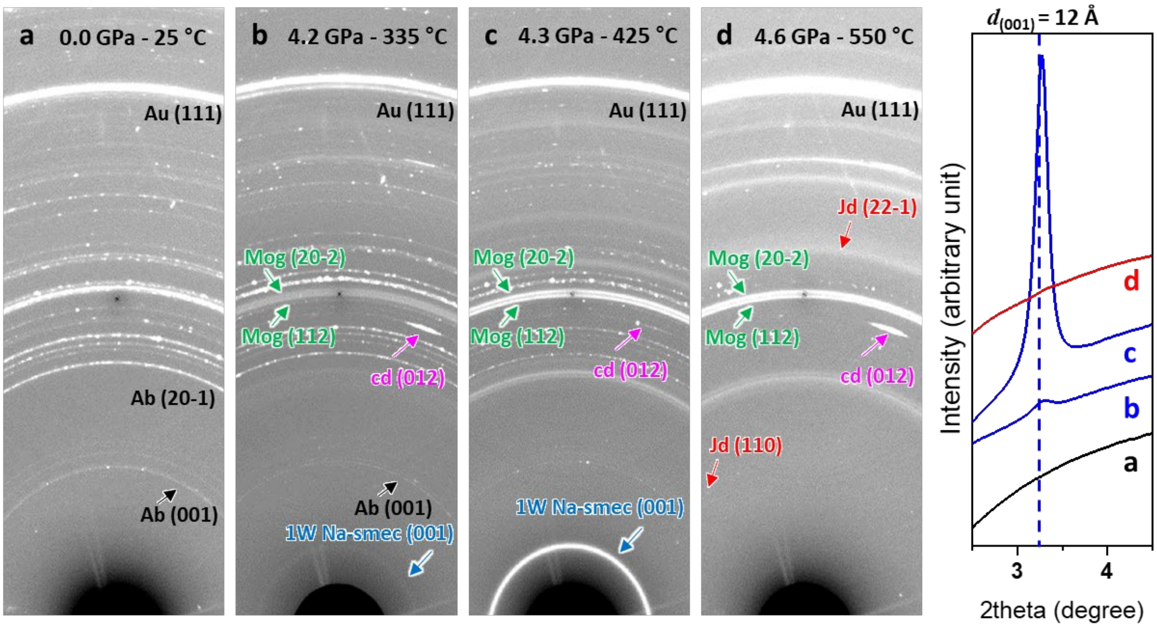

Supplementary Fig. 1 *In-situ* X-ray diffraction images showing the breakdown of albite at increasing PT conditions under water medium. **a,** Starting ambient conditions showing Debye-Scherrer rings from albite and Au pressure marker. **b,** PT conditions where the hydrated Na-smectite starts to form with (001) peak near 12 Å, which intensifies in **c**. The (001) peak of Na-smectite disappears and peaks from jadeite appear in **d.**

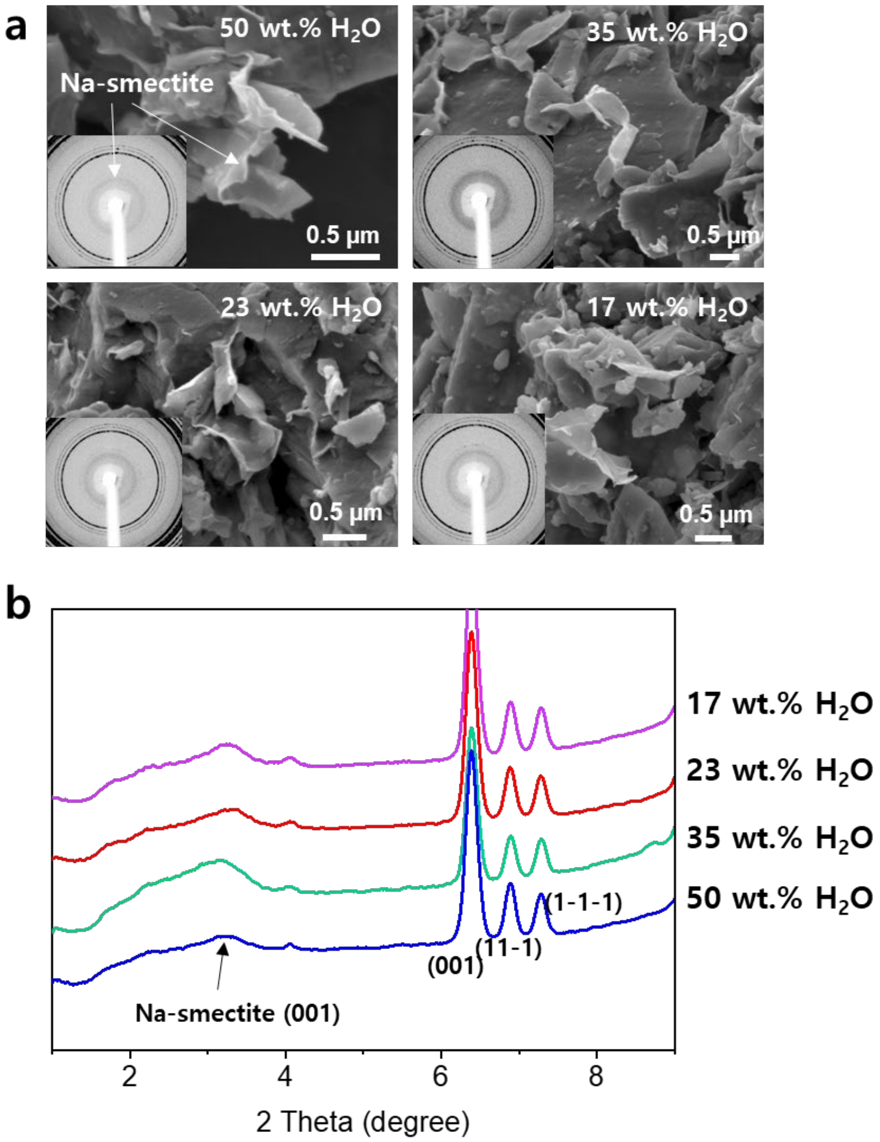

Supplementary Fig. 2 FE-SEM and X-ray diffraction images of the recovered products from the LVP experiments on albite at 2.4 GPa and 260 °C using different water contents. **a,** Characteristic clay crystal habits are seen in each recovered sample. Insets show respective diffraction images. **b,** XRD patterns show the formation of the hydrated smectite at $d_{001} \sim 12.7$ Å.

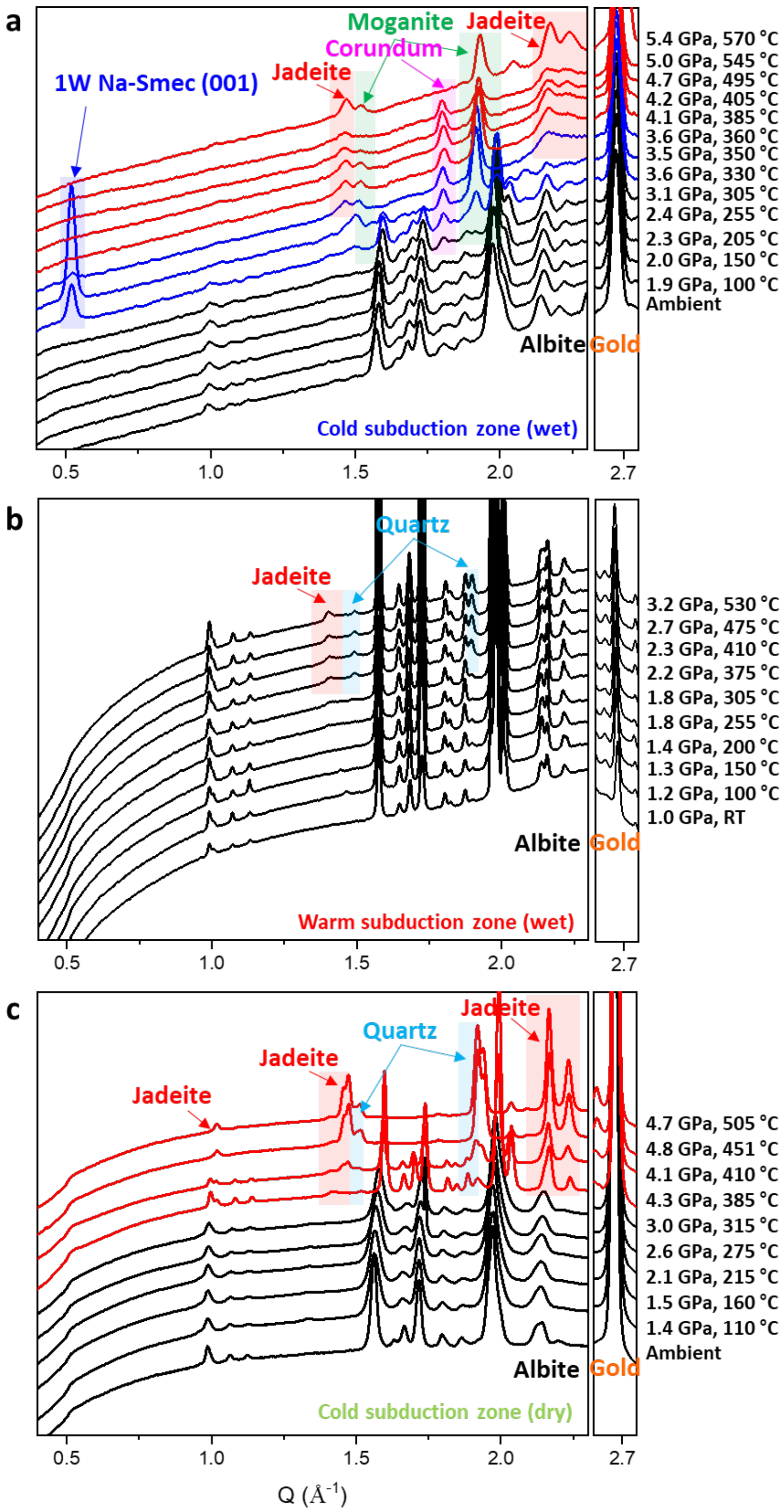

Supplementary Fig. 3 ***In-situ*** **X-ray diffraction patterns representing the stability of albite under three different PT conditions.** **a,** Under aqueous cold subduction conditions. **b,** Under aqueous warm subduction conditions. **c,** Under anhydrous cold subduction conditions. The gold peaks (right panel) were used as pressure scales.

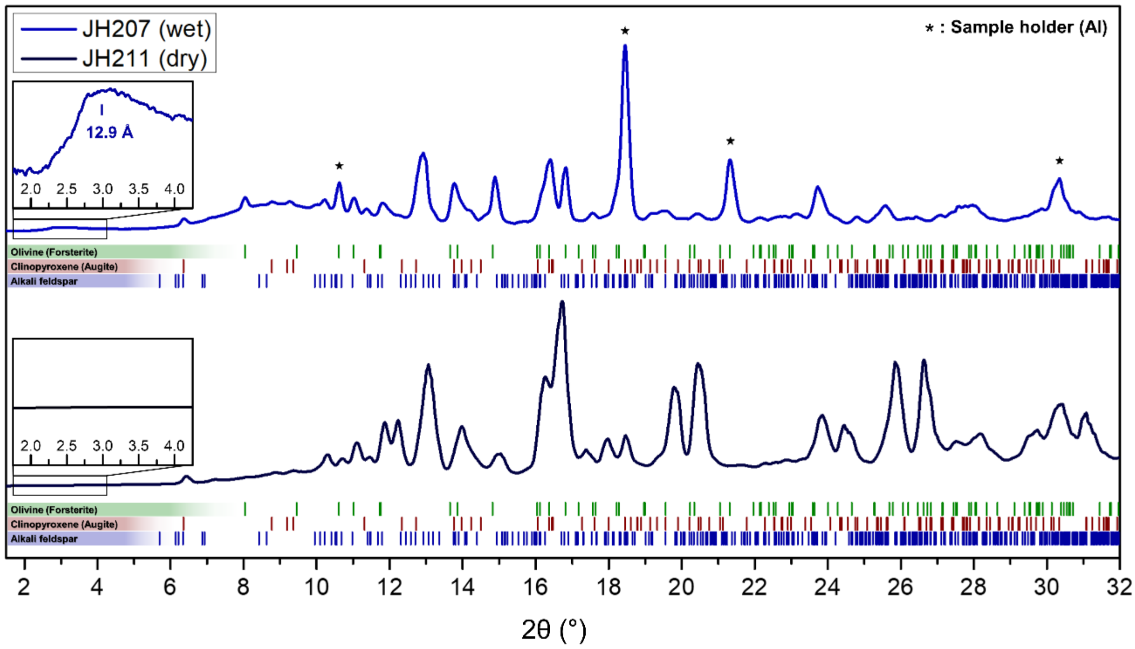

Supplementary Fig. 4 X-ray diffraction patterns of the recovered basalt rock sample after the modified Griggs apparatus experiment. The recovered basalt from 2.5 GPa and 250 ºC under wet condition by 5.5 wt.% H_2O (top). The recovered basalt from 2.5 GPa and 250 ºC under dry condition (bottom**).** Insets show the 2θ regions where d_{001} from the hydrated smectite is observed.

Supplementary Table 1 Calculated density of albite and its breakdown products at different PT conditions.

Pressure (GPa)	Temperature (ºC)	Phase	Density (*g cm*$^{-3}$)	Net crystalline density (*g cm*$^{-3}$) (Only albite)	Net crystalline density (*g cm*$^{-3}$) (Basalt with 6 wt.% H_2O)	Net fluid volume (Only albite)	Net fluid volume (Basalt with 6 wt.% H_2O)
0.0001	25	Albite	2.63(1)	2.63	3.16	1 m^3	1 m^3
0.8	25	Albite	2.66(1)	2.66	3.17		
1.2	60	Albite	2.59(1)	2.59	3.16		
1.2	110	Albite	2.72(1)	2.72	3.18		
2.8	285	Albite	2.58(1)	2.58	3.16		
2.9	290	Albite	2.58(1)	2.63	3.16	0.86 m^3	0.89 m^3
		Na-smectite (1W)	1.76(1)				
		Moganite	2.62(1)				
		Corundum	3.90(1)				
3.1	335	Albite	2.59(1)	2.63	3.16		
		Na-smectite (1W)	1.76(1)				
		Moganite	2.62(1)				
		Corundum	3.90(1)				
3.6	330	Albite	2.72(1)	2.70	3.18		
		Na-smectite (1W)	1.80(1)				
		Moganite	2.66(1)				
		Corundum	3.90(1)				
3.5	350	Albite	2.71(1)	2.69	3.17		
		Na-smectite (1W)	1.79(1)				
		Moganite	2.66(1)				
		Corundum	3.90(1)				
4.3	425	Albite	2.66(1)	2.68	3.17		
		Na-smectite (1W)	1.88(1)				
		Moganite	2.65(1)				
		Corundum	4.01(1)				
4.3	435	Jadeite	3.35(1)	2.98	3.22	0.93 m^3	0.94 m^3
		Moganite	2.77(1)				
		Corundum	3.97(1)				
4.6	550	Jadeite	3.33(1)	2.97	3.22		
		Moganite	2.68(1)				
		Corundum	4.03(1)				
5.0	545	Jadeite	3.36(1)	2.91	3.21		
		Moganite	2.68(1)				
		Corundum	3.90(1)				
5.4	570	Jadeite	3.36(1)	2.91	3.21		
		Moganite	2.68(1)				
		Corundum	3.90(1)				
